# O Pré-Condicionamento com Dexmedetomidina Atenua a Lesão de Isquemia/Reperfusão Miocárdica em Ratos, Suprimindo a Mitofagia Via Ativação do Receptor Α2-Adrenérgico

**DOI:** 10.36660/abc.20220750

**Published:** 2023-10-26

**Authors:** YaHua Chen, Hui Chen, YuJiao Chen, ZaiQun Yang, Tao Zhou, Wei Xu

**Affiliations:** 1 Guizhou Aerospace Hospital Zunyi Guizhou China Guizhou Aerospace Hospital, Zunyi, Guizhou – China; 2 Zunyi Medical University Zunyi Guizhou China Affiliated Hospital of Zunyi Medical University, Zunyi, Guizhou – China; 3 North Sichuan Medical College NanChong Sichuan China Affiliated Hospital of North Sichuan Medical College, NanChong, Sichuan – China; 4 People’s Hospital of Qiandongnan Miao Dong Autonomous Prefecture Qiandongnan Guizhou China People’s Hospital of Qiandongnan Miao and Dong Autonomous Prefecture, Qiandongnan, Guizhou – China

**Keywords:** Dexmedetomidina, Traumatismo por Reperfusão, Ratos, Mitofagia

## Abstract

**Fundamento:**

A dexmedetomidina (DEX), um agonista específico do receptor α2-adrenérgico, é protetora contra lesão de isquemia/reperfusão miocárdica (I/R). No entanto, a associação entre a cardioproteção induzida pelo pré-condicionamento DEX e a supressão da mitofagia permanece pouco clara.

**Objetivo:**

Portanto, nosso objetivo foi investigar se o pré-condicionamento com DEX alivia a I/R, suprimindo a mitofagia via ativação do receptor α2-adrenérgico.

**Método:**

Sessenta corações de ratos isolados foram tratados com ou sem DEX antes de induzir isquemia e reperfusão; um antagonista do receptor α2-adrenérgico, a ioimbina (YOH), também foi administrado antes da isquemia, isoladamente ou com DEX. A frequência cardíaca (FC), pressão diastólica do ventrículo esquerdo (PDVE), pressão diastólica final do ventrículo esquerdo (PDFVE), taxa máxima e mínima de desenvolvimento da pressão ventricular esquerda (±dp/dtmax) e tamanho do infarto do miocárdio foram medidos. A ultraestrutura mitocondrial e as autofagossomas foram avaliadas por microscopia eletrônica de transmissão. O potencial de membrana mitocondrial e os níveis de espécies reativas de oxigênio (ROS) foram medidos usando os ensaios JC-1 e diacetato de diclorodi hidrofluoresceína, respectivamente. Os níveis de expressão das proteínas associadas à mitofagia Beclin1, relação LC3II/I, p62, PINK1 e Parkin foram detectados por western blotting.

**Resultados:**

Em comparação com o grupo controle, no grupo isquemia/reperfusão, a FC, PDVE e ±dp/dtmax foram notavelmente diminuídas (p<0,05), enquanto os tamanhos da PDFVE e do infarto aumentaram significativamente (p<0,05). O pré-condicionamento com DEX melhorou significativamente a disfunção cardíaca, reduziu o tamanho do infarto do miocárdio, manteve a integridade estrutural mitocondrial, aumentou o potencial de membrana mitocondrial, inibiu a formação de autofagossomas e diminuiu a produção de ROS e a relação Beclin1, relação LC3II/I, expressão PINK1, Parkin e p62(p<0,05). Quando DEX e YOH foram combinados, o YOH cancelou o efeito da DEX, enquanto o uso de YOH sozinha não teve efeito.

**Conclusão:**

Portanto, o pré-condicionamento DEX foi cardioprotetor contra I/R em ratos, suprimindo a mitofagia por meio da ativação do receptor α2-adrenérgico.

## Figura Central

**Figure:**
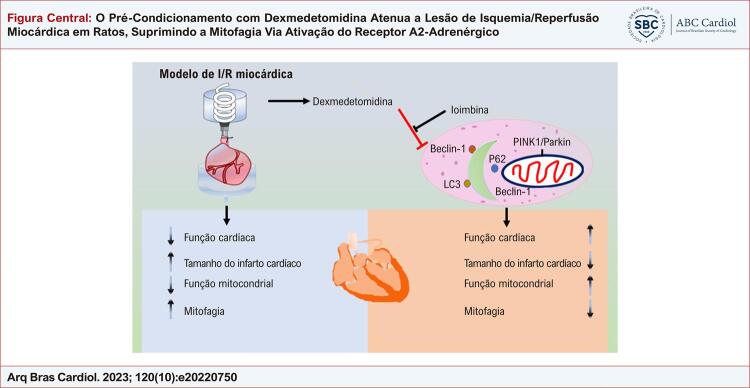
: O Pré-Condicionamento com Dexmedetomidina Atenua a Lesão de Isquemia/Reperfusão Miocárdica em Ratos, Suprimindo a Mitofagia Via Ativação do Receptor Α2-Adrenérgico

## Introdução

O infarto do miocárdio é um grande problema de saúde associado a alta morbidade e mortalidade em todo o mundo.^1^A melhor maneira de restaurar o miocárdio isquêmico é a reperfusão precoce para restaurar o fluxo sanguíneo e o suprimento de oxigênio à artéria coronária ocluída.^2^No entanto, além de reduzir a lesão isquêmica e limitar o tamanho do infarto, a reperfusão também pode causar independentemente uma lesão adicional chamada “lesão de reperfusão”.^3^Estudos experimentais sugeriram que uma lesão de reperfusão leva à perda da função cardíaca e à morte das células miocárdicas e contribui para até 50% do tamanho final do infarto.^4^Atualmente, nenhuma intervenção não farmacológica ou farmacológica eficaz está disponível para reduzir a lesão de isquemia/reperfusão miocárdica (I/R). Os mecanismos da I/R envolvem principalmente sobrecarga de cálcio, resposta inflamatória, estresse oxidativo, apoptose, disfunção mitocondrial e mitofagia,^5 - 13^ entre os quais a mitofagia desempenha um papel vital.

A autofagia é um processo de degradação relacionado ao lisossoma altamente conservado, ativado por hipoxemia, estresse oxidativo e organelas ou proteínas danificadas. É uma função de manutenção celular que remove organelas danificadas, incluindo as mitocôndrias, e mantém a sobrevivência celular.^14^A mitofagia, um tipo de autofagia seletiva, degrada mitocôndrias disfuncionais para manter uma rede mitocondrial normal e funcional.^15^As mitocôndrias são vulneráveis à lesão de isquemia/reperfusão (I/R), e as mitocôndrias danificadas são um elemento fisiopatológico do I/R.^16^Além disso, os cardiomiócitos são altamente dependentes da energia produzida pelas mitocôndrias e são mais sensíveis ao dano mitocondrial.^17^Assim, mais atenção está sendo dada à mitofagia devido à sua estreita correlação com o controle da quantidade e função mitocondrial durante a I/R. No entanto, a mitofagia pode ser excessivamente ativada após a reperfusão, resultando em diminuição da massa mitocondrial e agravamento da lesão cardíaca. Portanto, permanece controverso se a ativação da mitofagia é protetora ou prejudicial durante a I/R.

Dexmedetomidina (DEX), um agonista seletivo do adrenoceptor α2 (AR), é amplamente utilizado em anestesia cirúrgica e unidades de terapia intensiva devido ao seu efeito sedativo e à ausência de depressão respiratória.^18^Estudos anteriores sugeriram que o tratamento com DEX melhora os resultados cardíacos após cirurgia não cardíaca^19^ e reduz as taxas de mortalidade pós-operatória e complicações após cirurgia cardíaca humana. Estudos em animais também demonstraram que o pré-condicionamento com DEX protege contra lesões de I/R, reduzindo a incidência de arritmias ventriculares e a área de infarto.^20 , 21^Os atuais mecanismos moleculares da DEX têm se concentrado na inibição do estresse oxidativo, na redução da apoptose e na resposta inflamatória.^22 - 24^ No entanto, a associação entre DEX e mitofagia não está totalmente elucidada na I/R. Dada a importância da mitofagia na I/R, levantamos a hipótese de que o pré-condicionamento com DEX poderia suprimir a mitofagia após I/R em ratos, ativando os receptores α2-adrenérgicos.

Para testar nossa hipótese, estudamos 1) se o pré-condicionamento com DEX é cardioprotetor em um modelo animal de lesão I/R induzida pelo sistema de perfusão Langendorff e 2) se o efeito protetor é exercido por meio da supressão da mitofagia via a ativação de receptores α2-adrenérgicos.

## Métodos

### Animais

Nosso estudo aderiu às regras de pesquisa animal, revisadas e aprovadas por um comitê aprovado institucionalmente. Ratos Sprague-Dawley machos foram aprovados pelo Laboratory Animal Center da Tianjin Biological Company, Changsha, China (número do certificado: SYXK2019-0014). Todos os animais foram mantidos sob um ciclo claro/escuro de 12 horas a 22°C e receberam comida e água suficientes.

### Modelo de I/R miocárdica

Os ratos foram anestesiados com pentobarbital via injeção intraperitoneal (45 mg/kg). Posteriormente foi realizada toracotomia. Quando o coração foi exposto e a aorta cortada, o coração foi imediatamente isolado e imerso em solução nutritiva cardíaca gelada Krebs-Henseleit (KH) (NaCl, 119 mM; CaCl2, 1,24 mM; KCl, 6,0 mM; KH2PO4, NaHCO3, 20,1 mM; 1,24 mM; MgSO4, 1,24 mM; glicose, 11,2 mM). Foi então fixado no equipamento de perfusão Langendorff e perfundido com solução de KH mantida a uma pressão constante de 75–80 mmHg a 37°C. Um balão de látex cheio de água foi colocado no ventrículo esquerdo através da válvula mitral, e a outra extremidade do balão foi conectada a um transdutor de pressão para monitorar a função cardíaca, incluindo frequência cardíaca (FC), pressão diastólica do ventrículo esquerdo (PDVE), pressão diastólica final do ventrículo esquerdo (PDFVE), e a taxa máxima e mínima de desenvolvimento da pressão ventricular esquerda (±dp/dtmax).

### Grupos experimentais

**Figura 1:**
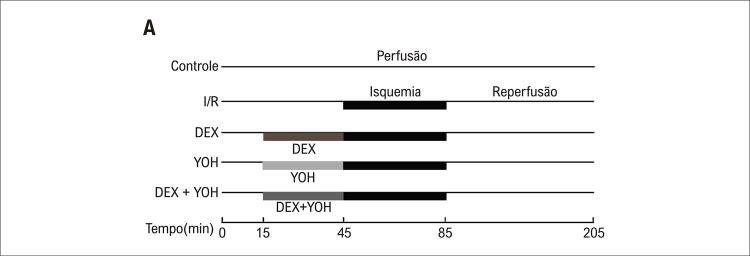
– Protocolo experimental para o coração de rato perfundido por Langendorff. Após um equilíbrio de perfusão de 15 minutos, os corações foram perfundidos durante 30 minutos adicionais e submetidos a 40 minutos de isquemia seguidos de 120 minutos de reperfusão. I/R: grupo isquemia/reperfusão; DEX: grupo Dexmedetomidina; YOH: grupo ioimbina.

Após um equilíbrio de 15 minutos, se o nível de PDVE e FC foram >80 mmHg e > 200 batimentos/min, respectivamente, os corações foram alocados aleatoriamente em cinco grupos (n = 12/grupo) por meio de uma randomização em envelope lacrado: o grupo controle (perfundido com solução de KH por 190 min), o grupo isquemia/reperfusão (I/R) (perfundido com solução de KH por 30 min seguido de 40 min de isquemia global e posterior reperfusão com solução de KH por 120 min), o grupo DEX (pré-condicionado com DEX 10nM por 30 min seguido de I/R), o grupo Ioimbina (YOH) (pré-condicionado com 1μM YOH por 30 min seguido de I/R), e o Grupo DEX + YOH (pré-condicionado com 10nM DEX + 1μM YOH por 30min seguido por I/R). Doses de DEX e YOH foram usadas conforme relatado anteriormente ( Figura1A ).^25^

### Coloração com cloreto de 2,3,5-trifeniltetrazólio (TTC)

A área do infarto foi avaliada pela coloração TTC. O coração foi colhido e congelado a -20°C ao final da reperfusão. Depois disso, todo o coração foi cortado em cinco seções iguais de cima para baixo e posteriormente incubado com TTC a 1% (Proteintech Group, Wuhan, China) por 30 min a 37°C no escuro. Finalmente, os corações foram fixados com formaldeído a 10% por 24 horas. As áreas não isquêmicas foram coradas de vermelho e as áreas infartadas foram coradas de branco. As áreas infartadas e normais foram fotografadas utilizando Image-Pro Plus 6.0. O tamanho do infarto foi calculado como uma porcentagem da área miocárdica total.

### Microscopia eletrônica de transmissão (MET)

O ventrículo esquerdo foi removido, cortado em formato de cubo de 1×1×1mm, rapidamente fixado com glutaraldeído a 2,5% por 24 horas e depois fixado com ósmio a 1% em temperatura ambiente por 2 horas. Posteriormente, o tecido foi lavado com solução salina tamponada com fosfato (PBS), desidratado em um gradiente de acetona e incorporado em uma mistura de resina epóxi. O tecido foi cortado em 50 fatias de nm de espessura usando um micrótomo manual Leica e depois duplamente corado com urânio e chumbo. A ultraestrutura e as autofagossomas no tecido miocárdico foram observadas e fotografadas usando um MET.

### Potencial de membrana mitocondrial (MMP)

As mitocôndrias foram extraídas do tecido ventricular esquerdo utilizando um Kit de Isolamento de Mitocôndrias de Tecido (Solarbio, Pequim, China) de acordo com as instruções do fabricante. Posteriormente, as mitocôndrias foram marcadas com JC-1 (Solarbio, Pequim, China), uma sonda fluorescente sensível. A fluorescência JC-1 foi detectada por microscopia de fluorescência. Mitocôndrias normais com alto potencial apresentaram fluorescência vermelha, enquanto mitocôndrias com baixo potencial exibiram fluorescência verde. Mudanças na despolarização mitocondrial foram refletidas na proporção das intensidades de fluorescência vermelha/verde.

### Produção de espécies reativas de oxigênio mitocondriais (ROS)

Para avaliar a produção de ROS, tecidos ventriculares esquerdos frescos e congelados foram incubados com solução de trabalho de diacetato de diclorodi hidrofluoresceína (DCFH-DA) (Servicebio, Wuhan, China) em PBS no escuro por 30 min a 37°C.

Após três lavagens com PBS, as seções foram continuamente incubadas com uma solução corante 4’,6-diamido-2-fenilindol (Servicebio, Wuhan, China) e seladas com um comprimido anti-fluorescência. As seções foram então observadas e analisadas usando um microscópio de fluorescência.

### Western blotting

O tecido cardíaco foi lisado com tampão de lisado proteico e o sobrenadante foi coletado. As concentrações de proteína foram medidas usando o ensaio de proteína do ácido bicinconínico (Servicebio, G2026). As amostras foram separadas por eletroforese em gel de poliacrilamida com dodecil-sulfato de sódio a 10% e transferidas para uma membrana de fluoreto de polivinilideno. Depois disso, a membrana foi bloqueada com leite desnatado a 5% por 30 min em temperatura ambiente e posteriormente incubada a 4°C por uma noite com os anticorpos primários, incluindo Beclin-1(Servicebio, GB112053, 1:1000), LC3 (Proteintech Grupo, 14600-1-AP, 1:1000), P62 (Servicebio, GB11239-1, 1:1000), PINK1 (Affinity, DF7742, 1:1000) e Parkin (Servicebio, GB11596, 1:1000) anticorpos policlonais de coelho. No dia seguinte, a membrana foi lavada com solução salina tamponada com Tris com tampão Tween e incubada por 30 min com anticorpos secundários (GB23303, Servicebio, 1:5000) em temperatura ambiente. O conteúdo destas proteínas foi determinado utilizando o software Alpha (alphaEaseFC, Alpha Innotech).

### Análise estatística

O tamanho da amostra deste estudo foi baseado no experimento preliminar. As médias da PDFVE no grupo controle, grupo DEX e grupo I/R após reperfusão por 120 minutos foram 12,52, 16,23 e 22,39, respectivamente, e os desvios padrão foram 1,45, 2,44 e 145, respectivamente. O teste do nível α foi considerado 0,05, Z0,05/2 = 1,96. O nível de poder, 1 − β, foi considerado 0,8. Para os grupos controle e DEX, foi necessário um tamanho de amostra de seis para cada grupo. Para os grupos I/R e DEX, foi necessário um tamanho de amostra de quatro para cada grupo. Assim, foi determinado um tamanho amostral de seis por grupo.

Os dados são expressos como média ± desvio padrão (DP). O software SPSS19.0 (International Business Machines, Corp) foi utilizado para análise estatística. A comparação dos dados de medição entre os grupos foi realizada utilizando ANOVA unidirecional. O teste Shapiro-Wilk foi empregado para verificar a normalidade. O método da Diferença Menos Significativa foi utilizado quando a variância era uniforme; de outra forma, foi utilizado o método T3 de Dunnett. A significância estatística foi estabelecida em p<0,05.

## Resultados

### O pré-condicionamento DEX melhorou a função cardíaca contra lesão de I/R

**Figura 2:**
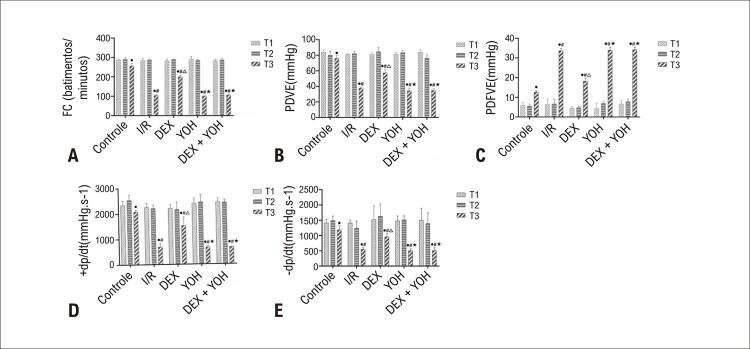
– Efeitos do T3 na lesão miocárdica mediada por I/R. (A-E): Efeitos cardioprotetores da DEX sobre FC, PDVE, PDFVE, +dp/dt e -dp/dt. Os dados foram apresentados como média ± desvio padrão. n=12.•p<0,05, vs. ponto inicial de isquemia, #p<0,05, vs. grupo controle na reperfusão por 120 minutos.∆p<0,05, vs. grupo I/R na reperfusão por 120 minutos. **#** p<0,05, vs. grupo DEX na reperfusão por 120 minutos. I/R: grupo isquemia/reperfusão; DEX: grupo Dexmedetomidina; YOH: grupo ioimbina. Painel A: FC: Frequência cardíaca. Painel B: PDVE: pressão diastólica do ventrículo esquerdo. Painel C: PDFVE: pressão diastólica final do ventrículo esquerdo. Painel D: +dp/dt: taxa máxima de desenvolvimento da pressão ventricular esquerda. Painel E: -dp/dt: taxa mínima de desenvolvimento de pressão ventricular esquerda.

Para investigar o efeito da DEX na função cardíaca, medimos os índices hemodinâmicos cardíacos em ratos com ou sem DEX. Nossos resultados mostraram que não houve diferenças significativas hemodinâmicas entre os grupos no início (T1) e no início da isquemia (T2) (p>0,05). Porém, ao final do período de reperfusão (T3), FC, PDVE,e ±dp/dtmax diminuíram e a PDFVE aumentou significativamente no grupo I/R comparado como grupo controle (p<0,05). O pré-condicionamento com DEX aumentou significativamente os valores de PDVE e ±dp/dtmax e diminuiu a PDFVE em comparação como grupo I/R (p<0,05). A adição de YOH reverteu bastante a melhora hemodinâmica alcançada pelo tratamento com DEX (p<0,05), enquanto a YOH sozinha não mostrou qualquer impacto na hemodinâmica (p>0,05) ( Figura 2 ).

### O pré-condicionamento com DEX reduziu o tamanho do infarto do miocárdio

**Figura 3:**
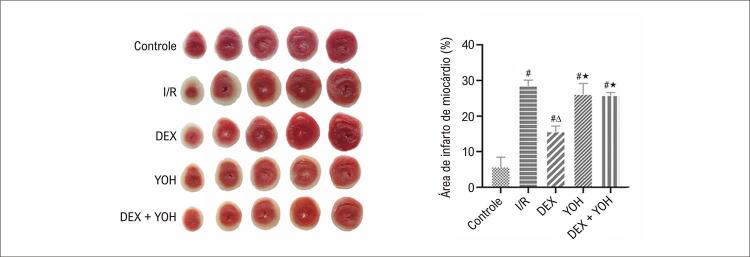
– DEX reduziu o tamanho do infarto cardíaco após lesão de I/R(n=6). Imagens representativas de amostras coradas com TTC mostrando a área de infarto (branco), a área sem infarto (vermelho) e a porcentagem de área infartada do coração no controle e coração isolado induzido por I/R. Os dados são apresentados como média ± desvio padrão, n=6. #p<0,05 νs. Grupo de controle; ∆P<0,05 νs. grupo I/R; ★p<0,05 νs. Grupo DEX. I/R: grupo isquemia/reperfusão; DEX: grupo Dexmedetomidina; YOH: Grupo ioimbina. TTC: técnica de coloração com cloreto de trifeniltetrazólio.

Para confirmar ainda que a DEX induz efeitos cardioprotetores contra o I/R, o tamanho do infarto foi determinado pela coloração com TTC. Como esperado, a I/R causou infarto do miocárdio significativo em comparação com o grupo controle (p<0,05). No entanto, o pré-condicionamento com DEX reduziu significativamente o tamanho do infarto em comparação como grupo I/R (p<0,05). A administração de YOH reverteu significativamente a diminuição no tamanho do infarto induzida por DEX (p<0,05). Além disso, comparado como grupo I/R, não houve diferenças entre os grupos YOH e DEX + YOH (p>0,05) ( Figura 3 ).

### O pré-condicionamento DEX reduziu a desordem e disfunção mitocondrial

**Figura 4:**
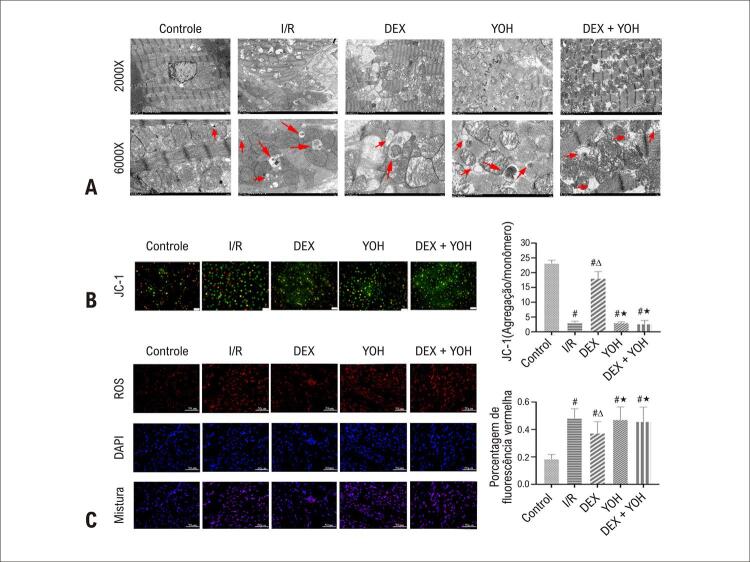
– DEX melhorou o distúrbio mitocondrial e a função mitocondrial. A) Imagens de microscopia eletrônica de transmissão de mitocôndrias (ampliação 2.000×, 6.000×): Setas vermelhas representam autofagossomas. B) Potencial de membrana mitocondrial detectado pela coloração JC-1 e a proporção da intensidade de mitocôndrias normais (fluorescência vermelha) /mitocôndrias hipopotenciais (fluorescência verde). Barra de escala: 100mm. C) ROS foi indicado pelo DCFH-DA, e os percentuais de intensidade de ROS (fluorescência vermelha) foram calculados em diferentes grupos, barra de escala: 50mm. Os dados são apresentados como média ± desvio padrão. n=6. #p<0,05 νs. Grupo de controle; ∆p<0,05 νs. grupo I/R; ★p<0,05 νs. Grupo DEX. I/R: grupo isquemia/reperfusão; DEX: grupo dexmedetomidina; YOH: Grupo ioimbina; ROS: Espécies reativas de oxigênio; DCFH-DA: diacetato diclorodi hidrofluoresceína.

Para explorar se a DEX melhorou a ultraestrutura mitocondrial, foi utilizado o MET. Os resultados mostraram que a I/R levou a danos mitocondriais acentuados, evidenciados por mitocôndrias inchadas e vacuolizadas com mais ruptura de cristas e ruptura de membrana mitocondrial em comparação com o grupo controle (p<0,05). Contrariamente, o dano mitocondrial foi mitigado e a integridade mitocondrial foi restaurada no grupo DEX em comparação com o grupo I/R (p<0,05). O co-tratamento com DEX e YOH reverteu significativamente o efeito protetor da DEX na ultraestrutura mitocondrial, o que foi equivalente ao do grupo I/R (p<0,05). A YOH sozinha não afetou as alterações ultraestruturais mitocondriais induzidas por I/R ( Figura 4A , p> 0,05).

Depois disso, examinamos a alteração no potencial de membrana mitocondrial usando o ensaio JC-1 para avaliar a função mitocondrial. Nossos resultados mostraram que a I/R induziu uma diminuição severa no potencial de membrana, aumentando significativamente a fluorescência verde em comparação com o grupo controle (p<0,05). No entanto, a DEX aliviou o declínio do potencial de membrana, que apareceu como maior relação de fluorescência vermelha/verde do que em I/R (p<0,05). Da mesma forma, este efeito pode ser bloqueado por YOH ( Figura 4B , p<0,05).

A solução de trabalho DCFH-DA foi usada para medir os níveis de ROS. Os resultados mostraram que os níveis de ROS estavam notavelmente elevados no grupo I/R comparado com o grupo de controle (p<0,05). O pré-condicionamento DEX reduziu significativamente a geração de ROS no grupo DEX (p<0,05). Da mesma forma, a YOH preveniu a inibição da produção de ROS induzida por DEX ( Figura 4C , p <0,05).

### O pré-condicionamento DEX suprimiu a mitofagia excessiva após I/R

**Figura 5:**
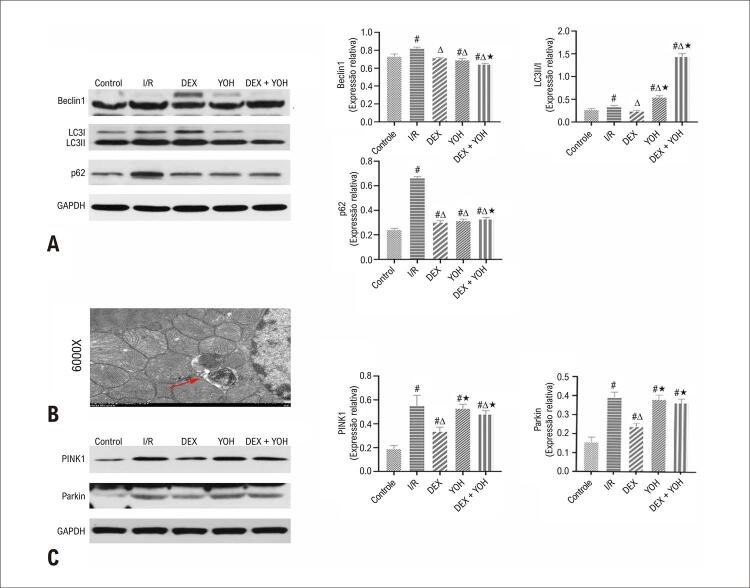
– Corações protegidos por DEX contra mitofagia excessiva induzida por I/R. A,C) Níveis de proteína de Beclin1, LC3II/I, p62, PINK1 e Parkin em diferentes grupos. B) Micrografia eletrônica representativa do coração obtida com aumento de 6.000 vezes. A seta vermelha indica autofagossoma engolindo mitocôndrias. Os dados são apresentados como média ± desvio padrão, n=6. #p<0,05 νs. Grupo de controle; ∆p<0,05 νs. grupo I/R; ★p<0,05 νs. Grupo DEX. I/R: grupo isquemia/reperfusão; DEX: grupo Dexmedetomidina; YOH: grupo Ioimbina; GAPDH: gliceraldeído-3-fosfato desidrogenase. Painel A: LC3: cadeia leve 3 da proteína 1 associada a microtúbulos, p62: Sequestossoma-1; Painel C: PINK1: suposta quinase 1 induzida por PTEN.

Para estudar ainda mais o mecanismo protetor da DEX na lesão I/R, marcadores bem conhecidos para proteínas relacionadas à formação de autofagossoma (relação Beclin1, LC3II/I) e proteínas de depuração de autofagossoma (p62) foram detectados por western blotting. Comparada com o grupo controle, a I/R aumentou os níveis da relação Beclin1 e LC3II/I, indicando aumento da formação de autofagossoma (p<0,05). O nível de p62 foi acentuadamente aumentado no grupo I/R em grau comparável ao do grupo controle (p<0,05). Comparado com o grupo I/R o pré-tratamento com DEX reduziu a expressão de Beclin1 e a relação LC3II/I (p<0,05), indicando que a DEX inibiu autofagia excessiva. Além disso, os níveis de proteína p62 diminuíram significativamente no grupo DEX comparado com o grupo I/R (p<0,05), indicando depuração eficiente do autofagossoma no grupo DEX. Consistentemente, a MET mostrou que a DEX reduziu o número de autofagossomas em comparação com o grupo I/R ( Figura 3A ). Contudo a YOH preveniu os efeitos da DEX na expressão Beclin1 LC3II/I e p62 ( Figura 5A , p<0,05), e no número de autofagossomas ( Figura 3A ).

Curiosamente, as imagens MET revelaram autofagossomas contendo mitocôndrias. Para explorar ainda mais o papel da DEX na autofagia, medimos a expressão de PINK1 e Parkin usando western blotting. Os resultados demonstraram que as proteínas PINK1 e Parkin estavam significativamente aumentadas no grupo I/R quando comparado com o grupo controle (p<0,05), e o pré-condicionamento com DEX inibiu a expressão de PINK1 e Parkin no grupo DEX (p<0,05). O tratamento com YOH reverteu o efeito inibitório da DEX na expressão de PINK1 e Parkin ( Figura 5C , p <0,05).

## Discussão

As descobertas deste estudo ampliam ainda mais nossa compreensão da DEX como um mecanismo de proteção contra a I/R. Primeiro, a DEX melhorou a função cardíaca, reduziu o tamanho do infarto do miocárdio e melhorou o comprometimento mitocondrial após I/R em modelo de rato in vitro. Além disso, o mecanismo protetor do pré-condicionamento de DEX pode envolver a supressão da mitofagia excessiva e a recuperação da depuração da autofagossoma, o que foi evidenciado pela inibição da formação da autofagossoma, pela diminuição de Beclin1, LC3II/I, PINK1 e Parkin, e pela redução de p62. No entanto, estes efeitos poderiam ser anulados pela adição de YOH. No geral, estes dados mostraram o papel protetor fundamental do pré-condicionamento de DEX na I/R, regulando a mitofagia através da ativação de α2-AR.

As mitocôndrias, como fonte de energia, desempenham um papel significativo no funcionamento e na sobrevivência dos cardiomiócitos.^26^Em nosso estudo, visualizamos mitocôndrias com microscopia eletrônica e mostramos que o pré-condicionamento DEX preservou a integridade mitocondrial, que foi mais semelhante ao grupo controle do que ao grupo I/R, em que as cristas mitocondriais foram obviamente rompidas. Também observamos um aumento no potencial de membrana mitocondrial e uma diminuição nas ROS mitocondriais no grupo DEX em comparação com aqueles no grupo I/R. As mitocôndrias normais são o principal local de produção de grandes quantidades de trifosfato de adenosina para as células e conduzem muitos processos biológicos.^27^No entanto, a estrutura e a função das mitocôndrias são complexas, variáveis e sensíveis. Assim, pequenas alterações nos fatores intracelulares ou extracelulares podem levar a anormalidades estruturais e disfunções mitocondriais. Além disso, estudos demonstraram que mitocôndrias danificadas e disfuncionais são a principal fonte de ROS intracelulares, que causam danos ao miocárdio.^28^ Além disso, a geração excessiva de ERO diminui o potencial da membrana mitocondrial, resultando em mitocôndrias mais danificadas. Acima de tudo, estas descobertas sugeriram que a remoção de mitocôndrias danificadas e a preservação da qualidade mitocondrial são a chave para proteger contra lesões de I/R e apoiaram ainda mais os papéis protetores críticos do pré-condicionamento de DEX nas mitocôndrias contra I/R.

Demonstrou-se que uma diminuição no potencial da membrana mitocondrial desencadeia a mitofagia após lesão de I/R.^29^ Portanto, determinamos se o efeito protetor da DEX nas mitocôndrias estava relacionado à regulação da mitofagia. A formação de autofagossoma é o padrão ouro para ativação de autofagia na mitofagia. Usando microscopia eletrônica, descobrimos que as autofagossomas eram mais abundantes no grupo I/R do que nos grupos DEX e de controle, e a imagem MET mostrou autofagossomas contendo mitocôndrias. Além disso, detectamos as proteínas conhecidas por desempenharem um papel fundamental na mitofagia, e os resultados mostraram que o pré-condicionamento com DEX reduziu Belin1, LC3II/I, PINK1, Parkin e a expressão da proteína p62 em comparação com o grupo I/R. As proteínas Beclin1 e LC3II/I facilitam a formação do autofagossoma e interagem com a p62 para mover as mitocôndrias danificadas para a autofagossoma.^30^ Sabe-se que as proteínas PINK1 e Parkin induzem mitofagia e limpam mitocôndrias excessivas e danificadas.^31^ Além disso, o acúmulo de autofagossomas, LC3II/I e p62 indica depuração prejudicada da autofagossoma.^32^ Assim, nossos dados sugeriram que a mitofagia foi excessivamente aumentada após I/R, enquanto o pré-condicionamento com DEX suprimiu a mitofagia excessiva e resgatou a depuração da autofagossoma para remover eficientemente as mitocôndrias danificadas. Zhang et al. reportaram que o bloqueio da depuração do autofagossoma causa acúmulo de autofagossoma e contribui à morte de cardiomiócitos.^33^ Nossos resultados apoiaram esse ponto de vista. A maioria dos estudos nesta área descreveram o papel protetor da mitofagia no infarto do miocárdio ou na lesão I/R.^34 , 35^ Em contraste com estes estudos, nossos resultados indicaram que a mitofagia foi excessivamente ativada e prejudicial ao coração após I/R. Em apoio às nossas descobertas, vários estudos anteriores mostraram que a mitofagia excessiva diminui as mitocôndrias, o que subsequentemente causa privação de energia e agrava a lesão miocárdica após I/R.^36 , 37^ Assim, o papel da mitofagia no I/R pode ser paradoxal.^38 , 39^ Finalmente, combinados com esses estudos, nossos achados sugeriram que altos níveis de atividade mitofágica e comprometimento da depuração da autofagossoma podem estar envolvidos no desenvolvimento de I/R em ratos. No entanto, a mitofagia leve induzida por DEX agiu como um mecanismo pró-sobrevivência para limpar eficientemente as mitocôndrias danificadas e prevenir I/R.

YOH, um bloqueador α2-AR, foi usado para explorar os efeitos da DEX no I/R. No presente estudo, a YOH reverteu os efeitos protetores do pré-tratamento com DEX no miocárdio de ratos, o que resultou em diminuição da função cardíaca e aumento do tamanho do infarto do miocárdio; além disso, os níveis de expressão das proteínas da via da mitofagia aumentaram. Juntos, esses resultados indicaram que o efeito protetor do pré-condicionamento com DEX no miocárdio de ratos pode ser antagonizado pelos bloqueadores α2-AR, o que é consistente com um estudo anterior.^40^ Os bloqueadores α2-AR podem dilatar o músculo liso vascular, diminuir o tônus simpático e aumentar o tônus parassimpático periférico . Uma investigação anterior de Okada et al. mostrou que a administração de DEX antes da isquemia e reperfusão global diminuiu o fluxo coronariano e o tamanho do infarto do miocárdio. Os autores propuseram que a vasoconstrição coronariana induzida por DEX pela estimulação de α2-AR diminuiu o fluxo coronariano, induziu isquemia miocárdica e desencadeou o pré-condicionamento cardíaco isquêmico. Assim, a DEX pode desempenhar um papel protetor no miocárdio, alterando o fluxo sanguíneo coronariano para induzir o pré-condicionamento isquêmico.^41^

Existem algumas limitações neste estudo. Não utilizamos agonistas da mitofagia ou uma tecnologia específica de nocaute genético para compreender completamente a atividade da mitofagia, embora esses métodos possam ser usados para confirmar definitivamente o mecanismo de proteção miocárdica da DEX na mitofagia. Portanto, mais estudos intensivos são necessários para esclarecer o mecanismo de proteção da DEX, que poderá fornecer suporte teórico para a aplicação da DEX no I/R.

## Conclusão

Este estudo apoiou a hipótese de que o pré-condicionamento com DEX exerceu um efeito protetor contra a I/R, suprimindo a mitofagia excessiva e limpando eficientemente as mitocôndrias danificadas ativando α2-AR, restaurando assim a função mitocondrial.
