# Supplementary material for: O Pré-Condicionamento com Dexmedetomidina Atenua a Lesão de Isquemia/Reperfusão Miocárdica em Ratos, Suprimindo a Mitofagia Via Ativação do Receptor Α2-Adrenérgico
Source: Arq Bras Cardiol. 2023 Oct 26;120(10):e20220750. [Article in Portuguese] doi: 10.36660/abc.20220750 (PMC12080598; doi:10.36660/abc.20220750)
Supplement: Translation

# Translation: Dexmedetomidine Preconditioning Attenuates Myocardial Ischemia/Reperfusion Injury in Rats by Suppressing Mitophagy Via Activating Α2-Adrenergic Receptor

**Authors:** YaHua Chen, Hui Chen, YuJiao Chen, ZaiQun Yang, Tao Zhou, Wei Xu

**Affiliations:** 1 China Guizhou Aerospace Hospital, Zunyi, Guizhou – China; 2 China Affiliated Hospital of Zunyi Medical University, Zunyi, Guizhou – China; 3 China Affiliated Hospital of North Sichuan Medical College, NanChong, Sichuan – China; 4 China People’s Hospital of Qiandongnan Miao and Dong Autonomous Prefecture, Qiandongnan, Guizhou – China

**Keywords:** Dexmedetomidine, Reperfusion Injury, Rats, Mitophagy

## Abstract

**Background:**

Dexmedetomidine (DEX), a specific α2-adrenergic receptor agonist, is protective against myocardial ischemia/reperfusion injury (MIRI). However, the association between DEX preconditioning-induced cardioprotection and mitophagy suppression remains unclear.

**Objective:**

Hence, we aimed to investigate whether DEX preconditioning alleviates MIRI by suppressing mitophagy via α2-adrenergic receptor activation.

**Method:**

Sixty isolated rat hearts were treated with or without DEX before inducing ischemia and reperfusion; an α2-adrenergic receptor antagonist, yohimbine (YOH), was also administered before ischemia, alone or with DEX. The heart rate (HR), left ventricular diastolic pressure (LVDP), left ventricular end-diastolic pressure (LVEDP), maximal and minimal rate of left ventricular pressure development (±dp/dtmax), and myocardial infarction size were measured. The mitochondrial ultrastructure and autophagosomes were assessed using transmission electron microscopy. Mitochondrial membrane potential and reactive oxygen species (ROS) levels were measured using JC-1 and dichloride hydrofluorescein diacetate assays, respectively. The expression levels of the mitophagy-associated proteins Beclin1, LC3II/I ratio, p62, PINK1, and Parkin were detected by western blotting.

**Results:**

Compared with the control group, in the ischemia/reperfusion group, the HR, LVDP, and ±dp/dtmax were remarkably decreased (p< 0.05), whereas LVEDP and infarct sizes were significantly increased (p< 0.05). DEX preconditioning significantly improved cardiac dysfunction reduced myocardial infarction size, maintained mitochondrial structural integrity, increased mitochondrial membrane potential, inhibited autophagosomes formation, and decreased ROS production and Beclin1, LC3II/I ratio, PINK1, Parkin, and p62 expression(p< 0.05). When DEX and YOH were combined, YOH canceled the effect of DEX, whereas the use of YOH alone had no effect.

**Conclusion:**

Therefore, DEX preconditioning was cardioprotective against MIRI in rats by suppressing mitophagy via α2-adrenergic receptor activation.

## Introduction

Myocardial infarction is a major health concern associated with high morbidity and mortality worldwide.^1^The best way to restore the ischemic myocardium is early reperfusion to restore blood flow and oxygen supply to the occluded coronary artery.^2^However, aside from reducing ischemic injury and limiting infarct size, reperfusion can also independently cause an additional injury called a “reperfusion injury”.^3^Experimental studies have suggested that a reperfusion injury leads to cardiac function loss and myocardial cell death and contributes to up to 50% of the final infarct size.^4^Currently, no effective non-pharmacological or pharmacological interventions are available to reduce myocardial ischemia/reperfusion injury (MIRI). The mechanisms of MIRI mainly involve calcium overload, inflammatory response, oxidative stress, apoptosis, mitochondrial dysfunction, and mitophagy,^5 - 13^among which mitophagy plays a vital role.

Autophagy is a highly conserved lysosomal-related degradation process activated by hypoxemia, oxidative stress, and damaged organelles or proteins. It is a cellular housekeeping function that removes damaged organelles, including the mitochondria and maintains cell survival.^14^Mitophagy, a type of selective autophagy, degrades dysfunctional mitochondria to maintain a normal and functional mitochondrial network.^15^Mitochondria are vulnerable to ischemia/reperfusion (I/R) injury, and damaged mitochondria are a pathophysiological element of MIRI.^16^Moreover, cardiomyocytes are highly dependent on the energy produced by the mitochondria and are more sensitive to mitochondrial damage.^17^Thus, more attention is being paid to mitophagy because of its close correlation with controlling mitochondrial quantity and function during MIRI. However, mitophagy may be excessively activated after reperfusion, resulting in decreased mitochondrial mass and aggravation of cardiac injury. Therefore, it remains controversial whether mitophagy activation is protective or detrimental during MIRI.

Dexmedetomidine(DEX), a selective α2-adrenoceptor(AR) agonist, is widely used in surgical anesthesia and intensive care units because of its sedative effect and lack of respiratory depression.^18^Previous studies have suggested that DEX treatment improves cardiac outcomes after non-cardiac surgery^19^and reduces postoperative mortality rates and complications after human cardiac surgery. Animal studies have also shown that DEX preconditioning protects against I/R injury by reducing the incidence of ventricular arrhythmias and the infarct area.^20 , 21^The current molecular mechanisms of DEX have focused on inhibiting oxidative stress, reducing apoptosis, and the inflammatory response.^22 - 24^However, the association between DEX and mitophagy is not fully elucidated in MIRI. Given the importance of mitophagy in I/R, we hypothesized that DEX preconditioning could suppress mitophagy after MIRI in rats by activating α2-adrenergic receptors.

To test our hypothesis, we studied 1) if DEX preconditioning is cardioprotective in an animal I/R injury model induced by the Langendorff perfusion system and 2) if the protective effect is exerted through the suppression of mitophagy via the activation of α2-adrenergic receptors.

## Methods

### Animals

Our study adhered to animal research rules as reviewed and approved by an institutionally approved committee. Male Sprague-Dawley rats were approved by the Laboratory Animal Center of Tianjin Biological Company, Changsha, China (certificate number: SYXK2019-0014). All animals were kept under a 12 h light/dark cycle at 22℃ and received sufficient food and water.

### Myocardial I/R model

The rats were anesthetized with pentobarbital via intraperitoneal injection (45 mg/ kg). Subsequently, a thoracotomy was performed. When the heart was exposed and the aorta was cut, the heart was immediately isolated and immersed in ice-cold cardiac nutrient solution Krebs-Henseleit (KH) (NaCl, 119 mM; CaCl2, 1.24 mM; KCl, 6.0 mM; KH2 PO4, NaHCO3, 20.1 mM; 1.24 mM; MgSO4, 1.24 mM; glucose, 11.2 mM). It was then fixed on the Langendorff perfusion setup and perfused with KH solution maintained at a constant pressure of 75–80 mmHg at 37℃. A latex balloon filled with water was placed in the left ventricle through the mitral valve, and the other end of the balloon was connected to a pressure transducer to monitor cardiac function, including heart rate (HR), left ventricular diastolic pressure (LVDP), left ventricular end-diastolic pressure (LVEDP), and the maximal and minimal rate of left ventricular pressure development (±dp/dtmax).

### Experimental groups

After a 15-minute equilibration, if the levels of LVDP and HR were > 80 mmHg and > 200 beats/min, respectively, the hearts were randomly allocated into five groups (n = 12/group) by a sealed envelope randomization assignment: the control group (perfused with KH solution for 190 min), the ischemia /reperfusion(I/R) group (perfused with KH solution for 30 min followed by 40 min of global ischemia and subsequent reperfusion with KH solution for 120 min), the DEX group (preconditioned with 10nM DEX for 30 min followed by I/R), the Yohimbine (YOH)group (preconditioned with1μM YOH for 30 min followed by I/R), and the DEX + YOH group (preconditioned with 10nM DEX + 1μM YOH for 30 min followed by I/R). Doses of DEX and YOH were used as previously reported ( Figure1A ).^25^

**Figure 1:** – Experimental protocol for the Langendorff perfused rat heart. After a 15-minute equilibration of perfusion, hearts were perfused for 30 additional minutes and subjected to 40 min of ischemia followed by 120 min of reperfusion. I/R: Ischemia/reperfusion group; DEX: Dexmedetomidine group; YOH: Yohimbine group.

### 2,3,5-Triphenyltetrazolium chloride (TTC) staining

The infarction area was assessed using TTC staining. The heart was harvested and frozen at −20℃ at the end of reperfusion. After that, the whole heart was cut into five equal sections from top to bottom and subsequently incubated with 1% TTC (Proteintech Group, Wuhan, China) for 30 min at 37°C in the dark. Finally, the hearts were fixed with 10% formaldehyde for 24 hours. Non-ischemic areas were stained red, and infarcted areas were stained white. The infarcted and normal areas were photographed using Image-Pro Plus 6.0. Infarct size was calculated as a percentage of the total myocardial area.

### Transmission electron microscopy (TEM)

The left ventricle was removed, cut into a 1×1×1 mm cube, rapidly fixed with 2.5% glutaraldehyde for 24 h, and then fixed with 1% osmium at room temperature for 2 h. Subsequently, the tissue was washed with phosphate-buffered saline (PBS), dehydrated in an acetone gradient, and embedded in an epoxy resin mixture. The tissue was cut into 50 nm thick slices using a manual Leica microtome and then double-stained with uranium and lead. The ultrastructure and autophagosomes in the myocardial tissue were observed and photographed using a TEM.

### Mitochondrial membrane potential (MMP)

Mitochondria were extracted from the left ventricular tissue using a Tissue Mitochondria Isolation Kit (Solarbio, Beijing, China) according to the manufacturer’s instructions. Subsequently, mitochondria were labeled with JC-1 (Solarbio, Beijing, China), a sensitive fluorescent probe. JC-1 fluorescence was detected by fluorescence microscopy. Normal mitochondria with a high potential showed red fluorescence, while mitochondria with a low potential exhibited green fluorescence. Changes in mitochondrial depolarization were reflected in the ratio of red/green fluorescence intensities.

### Mitochondrial reactive oxygen species (ROS) production

To evaluate the production of ROS, fresh and frozen left ventricular tissues were incubated with dichlorodi hydrofluorescein diacetate (DCFH-DA) working solution (Servicebio, Wuhan, China) in PBS in the dark for 30 min at 37°C. Following three washes with PBS, the sections were continuously incubated with a 4’,6-diamido-2-phenylindole (Servicebio, Wuhan, China) dye solution and sealed with an anti-fluorescence-quenched tablet. The sections were then observed and analyzed using a fluorescence microscope.

### Western blotting

The heart tissue was lysed with protein lysate buffer, and the supernatant was collected. Protein concentrations were measured using the bicinchoninic acid protein assay (Servicebio, G2026). The samples were separated using 10% sodium dodecyl-sulfate polyacrylamide gel electrophoresis and transferred to a polyvinylidene fluoride membrane. After that, the membrane was blocked with 5% skim milk for 30 min at room temperature and subsequently incubated at 4°C for a night with the primary antibodies, including Beclin-1(Servicebio, GB112053, 1:1000), LC3 (Proteintech Group, 14600-1-AP, 1:1000), P62 (Servicebio, GB11239-1, 1:1000), PINK1 (Affinity, DF7742, 1:1000), and Parkin (Servicebio, GB11596, 1:1000) rabbit polyclonal antibodies. The next day, the membrane was washed with Tris-buffered saline with Tween buffer and incubated for 30 min with secondary antibodies (GB23303, Servicebio, 1:5000) at room temperature. The content of these proteins was determined using Alpha (alphaEaseFC, Alpha Innotech) software.

### Statistical analysis

The sample size of this study was based on the preliminary experiment. Means of LVEDP in the control group, the DEX group, and the I/R group following reperfusion for 120 minutes were 12.52, 16.23, and 22.39, respectively, and standard deviations were 1.45, 2.44, and 145, respectively. The α-level test was considered as 0.05, Z0.05/2 = 1.96. The power level, 1 − β, was considered as 0.8. For the control and the DEX groups, a sample size of six was required for each group. For the I/R and the DEX groups, a sample size of four was required for each group. Thus, a sample size of six was determined per group.

Data are expressed as mean ± standard deviation (SD). SPSS19.0 (International Business Machines, Corp) software was used for statistical analysis. The comparison of measurement data between groups was carried out using one-way ANOVA. The Shapiro-Wilk test was employed to verify normality. The Least Significant Difference method was used when the variance was uniform; otherwise, Dunnett’s T3 method was used. Statistical significance was set at p< 0.05.

## Results

### DEX preconditioning improved cardiac function against I/R injury

To investigate the effect of DEX on cardiac function, we measured the cardiac hemodynamic indices in rats with or without DEX. Our results showed that there were no significant differences in hemodynamics between the groups at baseline (T1) and the beginning of ischemia (T2) (p>0.05). However, at the end of the reperfusion period (T3), HR, LVDP, and ±dp/dtmax were decreased, and LVEDP was significantly increased in the I/R group compared with the control group (p<0.05). DEX preconditioning significantly increased the LVDP and ±dp/dtmax values and decreased LVEDP compared with the I/R group (p<0.05). The addition of YOH greatly reversed the improvement in hemodynamics achieved by the DEX treatment (p<0.05), while YOH alone did not show any impact on hemodynamics (p>0.05) ( Figure 2 ).

**Figure 2:** – Effects of T3 on I/R-mediated myocardial injury. A-E) Cardioprotective effects of DEX on the HR, LVDP, LVEDP, +dp/dt, and -dp/ dt. Data were presented as the mean ± standard deviation. n=12. •p<0.05, vs. ischemia beginning point, #p<0.05, vs. control group at reperfusion for 120 minutes. ∆p<0.05, vs. I/R group at reperfusion for 120 minutes. #p<0.05, vs. DEX group at reperfusion for 120 minutes. I/R: Ischemia/reperfusion group; DEX: Dexmedetomidine group; YOH: Yohimbine group. Panel A: HR: Heart rate. Panel B: LVDP: left ventricular diastolic pressure. Panel C: LVEDP: left ventricular end-diastolic pressure. Panel D: +dp/dt: maximal left ventricular pressure development rate. Panel E: -dp/dt: minimal left ventricular pressure development rate.

### DEX preconditioning reduced the myocardial infarct size

To further confirm that DEX induces cardioprotective effects against MIRI, infarction size was determined by TTC staining. As expected, I/R caused significant myocardial infarction compared with the control group (p<0.05). However, DEX preconditioning significantly reduced infarct size compared to the I/R group (p<0.05). YOH administration significantly reversed the decrease in infarct size induced by DEX (p<0.05). Additionally, compared with the I/R group, there were no differences between the YOH and DEX + YOH groups (p>0.05) ( Figure 3 ).

**Figure 3:** – DEX reduced cardiac infarct size after I/R injury (n=6). Representative images of TTC stained samples showing the infarct area (white), the non-infarct area (red), and the infarcted area percentage of heart in control and I/R-induced isolated heart. Data are presented as the mean ± standard deviation, n=6. #p<0.05 νs. Control group; ∆p<0.05 νs. I/R group; ★p<0.05 νs. DEX group. I/R: Ischemia/reperfusion group; DEX: Dexmedetomidine group; YOH: Yohimbine group; TTC: triphenyltetrazolium chloride staining technique.

### DEX preconditioning reduced mitochondrial disorder and dysfunction

To explore whether DEX improved mitochondrial ultrastructure, TEM was used. The results showed that I/R led to marked mitochondrial damage, evidenced by swollen and vacuolated mitochondria with more cristae disruption and mitochondrial membrane rupture than the control group (p<0.05). Contrarily, mitochondrial damage was mitigated, and mitochondrial integrity was restored in the DEX group compared with the I/R group (p<0.05). Co-treatment with DEX and YOH significantly reversed the protective effect of DEX on the mitochondrial ultrastructure, which was equivalent to that in the I/R group (p<0.05). YOH alone did not affect the mitochondrial ultrastructural changes induced by I/R ( Figure 4A , p>0.05).

**Figure 4:** – DEX ameliorated mitochondrial disorder and improved mitochondrial function. A) Transmission electron microscopy images of mitochondria (magnification 2 000×, 6 000×): Red arrows represent autophagosomes. B) Mitochondrial membrane potential detected by JC-1 staining and the ratio of the normal mitochondria (red fluorescence)/hypopotential mitochondria (green fluorescence) intensity. Scale bar: 100mm. C) ROS was indicated by DCFH-DA, and the percentages of ROS (red fluorescence) intensity were calculated in different groups, scale bar: 50mm. Data are presented as the mean ± standard deviation. n=6. #p<0.05 νs. Control group; ∆p<0.05 νs. I/R group; ★p<0.05 νs. DEX group. I/R: Ischemia/reperfusion group; DEX: Dexmedetomidine group; YOH: Yohimbine group. Panel C: ROS: Reactive oxygen species; DCFH-DA: dichlorodi hydrofluorescein diacetate.

After that, we examined alterations in mitochondrial membrane potential using the JC-1 assay to assess mitochondrial function. Our results showed that I/R induced a severe decrease in membrane potential, significantly increasing green fluorescence compared with the control group (p<0.05). Nevertheless, DEX alleviated the decline in membrane potential, which appeared as a higher ratio of red/green fluorescence than that in I/R (p<0.05). Similarly, this effect can be blocked by YOH ( Figure 4B , p<0.05).

DCFH-DA working solution was used to measure ROS levels. The results showed that ROS levels were remarkably elevated in the I/R group compared with the control group (p<0.05). DEX preconditioning significantly reduced ROS generation in the DEX group (p<0.05). Similarly, YOH prevented DEX-induced inhibition of ROS production ( Figure 4C , p<0.05).

### DEX preconditioning suppressed the excessive mitophagy following I/R

To further study the protective mechanism of DEX in I/R injury, well-known markers for autophagosome formation-related proteins (Beclin1, LC3II/I ratio) and autophagosome clearance proteins (p62) were detected by western blotting. Compared with the control group, I/R increased the Beclin1 and LC3II/I ratio levels, indicating increased autophagosome formation (p<0.05). The level of p62 was markedly increased in the I/R group to a degree comparable to that in the control group (p<0.05). Compared with the I/R group, DEX pre-treatment reduced the expression of Beclin1 and the LC3II/I ratio (p<0.05), indicating that DEX inhibited excessive autophagy. Additionally, p62 protein levels were significantly decreased in the DEX group compared with the I/R group (p<0.05), indicating efficient autophagosome clearance in the DEX group. Consistently, TEM showed that DEX reduced the number of autophagosomes compared with that in the I/R group ( Figure 3A ). However, YOH prevented the effects of DEX on Beclin1, LC3II/I and p62 expression ( Figure 5A , p<0.05), and the number of autophagosomes ( Figure 3A ).

**Figure 5:** – DEX protected hearts against I/R-induced excessive mitophagy. A, C) Protein levels of Beclin1, LC3II/I, p62, PINK1, and Parkin in different groups. B) Representative electron micrograph of the heart obtained at a magnification of 6000 times. The red arrow indicates autophagosome engulfing mitochondria. Data are presented as the mean ± standard deviation, n=6. #p<0.05 νs. Control group; ∆p<0.05 νs. I/R group; ★p<0.05 νs. DEX group. I/R: Ischemia/reperfusion group; DEX: Dexmedetomidine group; YOH: Yohimbine group; GAPDH: glyceraldehyde-3-phosphate dehydrogenase. Panel A: LC3: microtubule-associated protein 1 light chain 3, p62: Sequestosome-1; Panel C: PINK1: PTEN-induced putative kinase 1.

Interestingly, TEM images revealed autophagosomes containing mitochondria. To further explore the role of DEX in autophagy, we measured the expression of PINK1 and Parkin using western blotting. The results demonstrated that PINK1 and Parkin proteins were significantly increased in the I/R group when compared with the control group (p<0.05), and DEX preconditioning inhibited the expression of PINK1 and Parkin in the DEX group (p<0.05). Treatment with YOH reversed the inhibitory effect of DEX on PINK1 and Parkin expression ( Figure 5C , p<0.05).

## Discussion

The findings of this study further extend our understanding of DEX as a protective mechanism against MIRI. First, DEX improved cardiac function, reduced myocardial infarction size, and ameliorated mitochondrial impairment following MIRI in a vitro rat model. In addition, the protective mechanism of DEX preconditioning may involve suppressing excessive mitophagy and rescoring autophagosome clearance, which was evidenced by the inhibition of autophagosome formation, the decrease of Beclin1, LC3II/I, PINK1, and Parkin, and by reducing p62. However, these effects could be canceled by the addition of YOH. Overall, these data showed the pivotal protective role of DEX preconditioning in MIRI by regulating mitophagy through activating α2- AR.

Mitochondria, as a source of energy, play a significant role in the functioning and survival of cardiomyocytes.^26^In our study, we imaged mitochondria with electron microscopy and showed that DEX preconditioning preserved mitochondrial integrity, which was more similar to the control group than the I/R group, wherein mitochondrial cristae were obviously disrupted. We also observed an increase in mitochondrial membrane potential and a decrease in mitochondrial ROS in the DEX group compared with those in the I/R group. Normal mitochondria are the main site for producing large quantities of adenosine triphosphate for cells and drive many biological processes.^27^However, the structure and function of mitochondria are complex, variable, and sensitive. Thus, slight changes in intracellular or extracellular factors can lead to structural abnormalities and mitochondrial malfunctions. In addition, studies have demonstrated that damaged and dysfunctional mitochondria are the major source of intracellular ROS, which causes myocardial damage.^28^Moreover, excessive ROS generation decreases mitochondrial membrane potential, resulting in more damaged mitochondria. Above all, these findings suggested that removing damaged mitochondria and preserving mitochondrial quality is the key to protecting against I/R injury and further supported the critical protective roles of DEX preconditioning on mitochondria against MIRI.

A decrease in mitochondrial membrane potential has been shown to trigger mitophagy after I/R injury.^29^Therefore, we determined whether the protective effect of DEX on mitochondria was related to the regulation of mitophagy. Autophagosome formation is the gold standard for autophagy activation in mitophagy. Using electron microscopy, we found that autophagosomes were more abundant in the I/R group than in the DEX and control groups, and the TEM image showed autophagosomes containing mitochondria. Furthermore, we detected the proteins known to play a key role in mitophagy, and the results showed that DEX preconditioning reduced Belin1, LC3II/I, PINK1, Parkin, and p62 protein expression compared with the I/R group. Beclin1 and LC3II/I proteins facilitate autophagosome formation and interact with p62 to move damaged mitochondria to the autophagosome.^30^PINK1 and Parkin proteins are known to induce mitophagy and clear excessive and damaged mitochondria.^31^In addition, the accumulation of autophagosomes, LC3II/I, and p62 indicates impaired autophagosome clearance.^32^Thus, our data suggested that mitophagy was excessively enhanced after I/R, whereas DEX preconditioning suppressed excessive mitophagy and rescored autophagosome clearance to remove damaged mitochondria efficiently. Zhang et al. reported that the blockade of autophagosome clearance causes autophagosome accumulation and contributes to cardiomyocyte death.^33^Our results supported this standpoint. Most studies in this area have described the protective role of mitophagy on myocardial infarction or I/R injury.^34 , 35^In contrast to these studies, our results indicated that mitophagy was excessively activated and detrimental to the heart after I/R. In support of our findings, several previous studies have shown that excessive mitophagy decreases mitochondria, which subsequently causes energy deprivation and aggravates myocardial injury following I/R.^36 , 37^Thus, the role of mitophagy in MIRI may be paradoxical.^38 , 39^Finally, combined with these studies, our findings suggested that high levels of mitophagy activity and impaired autophagosome clearance may be involved in developing MIRI in rats. However, DEX-induced mild mitophagy acted as a pro-survival mechanism to efficiently clear damaged mitochondria and prevent MIRI.

YOH, an α2-AR blocker, was used to explore the effects of DEX on MIRI. In the present study, YOH reversed the protective effects of DEX pre-treatment in the myocardium of rats, which resulted in decreased cardiac function and increased myocardial infarct size; in addition, the expression levels of the mitophagy pathway proteins were increased. Together, these results indicated that the protective effect of DEX preconditioning in rat myocardium may be antagonized by α2- AR blockers, which is consistent with a previous study.^40^α2-AR blockers can dilate vascular smooth muscle, decrease sympathetic tone, and increase peripheral parasympathetic tone. A previous investigation by Okada et al. showed that DEX administration prior to global ischemia and reperfusion decreased coronary flow and myocardial infarct size. The authors proposed DEX-induced coronary vasoconstriction by α2-AR stimulation decreased coronary flow, induced myocardial ischemia, and triggered ischemic heart preconditioning. Thus, DEX may play a protective role in the myocardium by altering coronary blood flow to induce ischemic preconditioning.^41^

There are some limitations in this study. We did not use mitophagy agonists or a specific gene knockout technology to fully understand mitophagy activity, though these methods could be used to confirm the myocardial protective mechanism of DEX in mitophagy definitively. Therefore, further intensive studies are required to clarify the protective mechanisms of DEX, which may provide theoretical support for the application of DEX in MIRI.

## Conclusion

This study supported the hypothesis that DEX preconditioning exerted a protective effect against MIRI by suppressing excessive mitophagy and efficiently clearing damaged mitochondria by activating α2-AR, thereby restoring mitochondrial function.
